# Improvement Science in Anaesthesia

**DOI:** 10.1007/s40140-017-0234-5

**Published:** 2017-09-23

**Authors:** Duncan T. Wagstaff, James Bedford, S. Ramani Moonesinghe

**Affiliations:** 1 UCL/UCLH Surgical Outcome Research Centre (SOuRCe), 3rd Floor, Maple Link Corridor, University College Hospital, 235 Euston Road, London, NW1 2BU UK; 2 National Institute of Academic Anaesthesia Health Services Research Centre (NIAA HSRC), Royal College of Anaesthetists, Churchill House, 35 Red Lion Square, London, WC1R 4SG UK; 3 Department of Applied Health Research (DAHR), University College London, 1–19 Torrington Place, London, WC1E 7HB UK

**Keywords:** Improvement Science, Anaesthesia, Quality, Data feedback

## Abstract

**Purpose of Review:**

This article offers an overview of the history and features of Improvement Science in general and some of its applications to Anaesthesia in particular.

**Recent Findings:**

Improvement Science is an evolving discipline aiming to generate learning from quality improvement interventions. An increasingly common approach to improving Anaesthesia services is to employ large-scale perioperative data measurement and feedback programmes. Improvement Science offers important insights on questions such as which indicators to collect data for; how to capture that data; how it can be presented in engaging visual formats; how it could/should be fed back to frontline staff and how they can be supported in their use of data to generate improvement.

**Summary:**

Data measurement and feedback systems represent opportunities for anaesthetists to work with multidisciplinary colleagues to help improve services and outcomes for surgical patients. Improvement Science can help evaluate which approaches work, and in which contexts, and is therefore of value to healthcare commissioners, providers and patients.

## Introduction

Improvement Science is an emerging concept which can be considered the scientific underpinning of quality improvement (QI) exploring how it can be best be undertaken [1•]. It uses rigorous scientific methods to understand and evaluate the QI process. The overriding goal of Improvement Science is to ensure that QI efforts are based as much on evidence as the best practices they seek to implement [2].

Anaesthesia is a rich environment for applying the methods of Improvement Science. Anaesthetists are characteristically numerate, accustomed to viewing data as time series and can understand the applications (and limitations) of representing complex systems with quantitative indicators. Increasingly, perioperative process and outcome measures are collected by large-scale programmes and fed back to hospitals with the hope of reducing variation in quality and driving system-wide improvement. Understanding the best ways of collecting, analysing, visualising, feeding back and supporting the use of this type of data is an important application of Improvement Science.

This review describes the historical development of Improvement Science as a discipline and how it is being used in Anaesthesia. It then focusses in detail on what contributions it can make to large-scale programmes aiming to improve quality by collecting and feeding back data.

## What Are the Features of Improvement Science?

There is currently an absence of an agreed definition of Improvement Science. It sits within the wider arena of health services research, and readers would be forgiven for confusing it with constituent or overlapping academic disciplines such as implementation science, translational science, measurement for improvement, quality improvement science, science of quality improvement, evidence-based practice and knowledge translation [1•]. To complicate matters further, terminology may vary internationally: In the USA, ‘improvement science’ has been used to describe structured quality improvement methods such as PDSA cycles or data interrogation [3, 4].

The field is predominantly concerned with healthcare, but manufacturing industries, aviation, software development, the military and other similar sectors have also systematically explored the most effective ways to improve quality and efficiency [5•]. The first steps in Improvement Science can be attributed to W Edwards Deming, an American statistician and business consultant. He originally designed an approach to reduce variation in industrial processes in the Japanese automotive industry and was later credited with rescuing the fortunes of Ford Motor Company in the 1980s. He devised a System of Profound Knowledge, through which we should view the world through four lenses: the system; the nature of variation; psychology of stakeholders and epistemology (the theory of truth/knowledge). By developing the concept of statistical process control, Deming helped companies understand and improve the quality of their systems and reap associated benefits in terms of cost reductions and productivity.

These principles have been applied to tackle deficiencies in healthcare processes such as unscientific care; inappropriate care; geographic variations in practice; latent disagreements between clinicians and unrecognised medical injuries to patients [5•]. QI typically uses tools such as process-mapping; measurement; plan-do-study-act (PDSA) cycles (also known as the model for improvement) and data visualisation [6]. Example categories of QI interventions include bundles; checklists; audit and feedback and innovative clinical services [6].

However, whilst Deming’s statistical process control approach may be valid for conducting QI in industrial settings, it does not take account of the complexity of the challenges facing healthcare. Furthermore, it may be too narrow as a definition of Improvement Science as it underplays the importance of robust assessment of improvement projects [7••]. A Health Foundation review noted that ‘whereas “improvement” focuses on optimising the benefits of change, “improvement science” focuses on maximising learning from improvement’ [1•]. Marshall emphasises that rigorous evaluation is needed to maximise the effectiveness and reduce the risks of QI, such as wasted time and resources, ignorance of side-effects and lack of evidence of positive change [7••].

Evaluating QI efforts in complex healthcare scenarios demands new types of studies, different to the scientific methods used to establish other types of medical evidence. A broad range of techniques is required, in a similar way to how engineering draws upon different disciplines to make practical changes to real-world problems [7••]. The choice of method(s) to answer any research question is pragmatic and includes innovative methods such as stepped wedge random controlled trials (used to evaluate the EPOCH study discussed below) and ethnographic studies.

Don Berwick has called for analyses to use a context-mechanism-outcome (CMO) approach first described by Pawson and Tilley [5•, 8]. This approach describes improvement programs as introducing new ideas to teams which may or may not take them up depending on their local context. As an example, it could help to explain why MERIT, a cluster randomised trial of rapid response teams, found no ‘evidence’ of benefit of what was locally reported as a good idea. Berwick argues that not only was the trial underpowered and compromised by cross-contamination, but there were also underlying flaws with the epistemological basis of the trial, and that among the ‘changing terrain of leadership, details of implementation and organisational factors…the RCT is an impoverished way to learn. Critics who use it as truth standard in this context are incorrect’ [5•].

In contrast to the necessarily local focus of QI, Improvement Science aims to create generalizable knowledge of sufficient rigour to be published in respected peer-reviewed academic journals. It aims to not only analyse which QI efforts ‘work’ but also to examine the best ways to measure and disseminate this learning in order to enable replication and spread of success [1•]. Greenhalgh took this concept further by using the results from a systematic review to derive a conceptual model of ‘diffusion of innovation’, describing the many linkages between the innovation and its contexts [9].

Understanding how interventions work (or do not) is essential to facilitate effective scaling and replication. To do this, a hypothesis (‘theory’) is needed which can then be tested against empirical evidence, and then subsequently accepted, rejected or refined. Confusingly, many terms are often used interchangeably to describe these hypotheses of how interventions work. Logic models tend to be descriptive, whereas theories of change aim to explain why interventions achieve the results that they do [10]. For example, an improvement programme to reduce central line infections in Michigan by employing a technical and cultural intervention was accompanied by appropriate measurement. The well-articulated theory of change could therefore be tested and used to inform implementation of the intervention elsewhere [11]. Both logic models and theories of change are examples of programme theories, which are limited in scale to the intervention being studied rather looking at wider levels of abstraction [12•].

Various ‘objective’ frameworks have been derived to help predict which innovations will be successful in which contexts. The ‘Promoting Action on Research Implementation in Health Services’ framework (PARIHS) considers the key elements influencing successful implementation of evidence-based practices [13]. Similarly, in the USA, a Consolidated Framework For Implementation Research (CFIR) has been developed [14]. The framework includes five major domains: intervention characteristics, outer setting, inner setting, characteristics of the individuals involved and the process of implementation. The Model for Understanding Success in Quality (MUSIQ) is a ‘conceptual model to help organisations and QI researchers understand and optimise contextual factors affecting the success of QI projects’ [15].

A genuine partnership between clinicians and academics is required to achieve Improvement Science’s ambition of creating practical learning that can make a timely difference to patient care. Clinicians can bring contextual and subject knowledge, whereas academics can contribute scepticism and methodological rigour. Positioning Improvement Science between research and audit, the Health Foundation suggests that ‘Improvement Science describes how to reduce the gap between what is actual (i.e. audit) and what is possible (i.e. research)’.

### Applications of Improvement Science in Anaesthesia

Anaesthetists are well placed to lead improvement projects within hospitals due to their multidisciplinary skill sets and working practices. They tend to have strong numerical skills and routinely examine data for trends in clinical settings. They have been becoming increasingly familiar with a safety culture, surgical checklists and care bundles over the last 10 years. In the UK, the Royal College of Anaesthetists (RCoA) has incorporated aspects of QI into the training curriculum, and trainees are expected to complete an audit or QI project every year.

Improvement Science is starting to gain traction in the field of Anaesthesia. One interesting example is the spread of large-scale monitoring and feedback programmes such as the National Surgical Quality Improvement Project (NSQIP), led by the American College of Surgeons. A systematic review of the benefits associated with participating in NSQIP reported that mere involvement in the programme reduced surgical morbidity although these advantages were greatest when formal QI programs were implemented [16]. This dataset has now been used to produce over 700 academic publications.

In the UK, the National Emergency Laparotomy Audit (NELA) has now been running for 3 years. Superimposed upon this platform for collecting and feeding back data have been several successful initiatives, such as the Emergency Laparotomy Collaborative and EPOCH (described below). Going forward, the Perioperative Quality Improvement Programme (PQIP) led by the RCoA and its Health Services Research Centre will prospectively collect process and outcome data for major elective surgery across the UK. It aims to use Improvement Science methods to feedback data in near real-time with appropriate support to enable hospitals to drive local quality improvement.

Context clearly affects the success or failure of an improvement project in Anaesthesia as in any other specialty. For example, one qualitative study reported on the differences in compliance with the WHO safer surgery checklist between high and low income settings. They reported that some resentment of the checklist was present, especially where there was conflict between the underlying philosophy of the checklist and local social, cultural and economic contexts. Understanding these contexts may therefore help predict and optimise implementation of improvement projects [17]. Much further work has gone on to classify context, for example at macro (national/regional), meso (organisational) and micro (team/department) levels [18].

## Measurement for Improvement

A recurring theme of Improvement Science is the concept for measuring for improvement, which we discuss further here.

### Data Capture

The development of electronic health records (EHR) over the past 10–15 years has provided large datasets that can be used to support quality improvement. By 2014, over 80% of non-federal acute care hospitals across the USA had adopted at least a basic EHR system [19]. A basic EHR was defined as system that allowed electronic recording and access to patient demographics, problem lists, medication lists, discharge summaries and diagnostic test results. In the UK, there are plans to make the NHS ‘largely paperless’ by 2020 [20]. This increase in EHR adoption allows the use of routine data to provide baseline performance and ongoing monitoring of quality improvement initiatives. Etzioni et al. published work assessing the impact of the American College of Surgeons National Quality Improvement Program (NSQIP), this included analysis of 345,357 hospitalisations in 113 different academic institutions [21•]. As well as institutional clinical datasets, there are also national administrative datasets such as hospital episode statistics (HES) in the UK, which is controlled by NHS Digital. Linking to such datasets can provide additional information and reduce the burden of data collection for local teams. In the UK, this approach has been taken by national projects such as the NELA and the recently launched PQIP which are both run by the National Institute of Academic Anaesthesia’s Health Services Research Centre based at the Royal College of Anaesthetists.

The concept of ‘big data’ in healthcare research is relatively new, and its increasing use is directly related to the adoption of EHRs. The term ‘big data’ is a fluid term that is dependent upon the interpretation of the user but involves five concepts of volume, variety, velocity, value and veracity [22]. By using electronic health records, electronic data capture, and linking to existing national datasets, all the five Vs can be increased.

### Data Quality Control

Of course, there are potential problems with using large datasets and electronic health records for quality improvement. The accuracy of data entered in to them directly influences the validity of any findings, and where routine datasets are used, the quality of this cannot always be assured. Quality control methods, such as those highlighted in Table 1, are critical to help ensure the accuracy of any effort to collect, analyse and report data [23•].

**Table 1:** Data quality control methods for QI projects. Reproduced from Needham DM, Sinopoli DJ, Dinglas VD, Berenholtz SM, Korupolu R, Watson SR, et al. Improving data quality control in quality improvement projects. Int. J. Qual. Heal. Care. 2009;21:145–50, with permission from Oxford University Press

Project phase	Challenge question
Project design	Are the aims of the project clearly stated? Is a valid definition and measurement system available for the required data? Is there a clear focus on quality, rather than quantity, of data?
Data collection	Is a standardised data collection form created? Are data items clearly defined and written instructions provided for collecting each data item? Are staff adequately trained to collect data? Are QA reviews completed? Is an electronic database used for data management? Are sufficient database controls in place to identify errors? Is there a back-up routine for the electronic database?
Data management	Have data been evaluated using basic statistics? Has there been a comprehensive review for missing data and methods to minimise missing data?
Data analysis	Are missing data reported and appropriate methods used to account for it? Have potential outliers been identified and evaluated? Have appropriate methods been used to provide summary measures of the project results? Have measures of precision been presented with the study results? Have appropriate methods been used to evaluate the impact of factors that may confound the results?

By considering data quality control throughout the entire design of an improvement project investigators will be able to improve the face validity of their findings. In return, this should improve uptake of results and adoption of change processes.

### What to Measure: Process vs. Outcome

The Darzi report [24], which was a seminal report on the future of the NHS in England published in 2008, stressed the need for a more positive approach to measurement for quality improvement. ‘In order to work out how to improve we need to measure and understand exactly what we do. The NHS needs a quality measurement framework at every level.’

Clinical trials almost unanimously use outcome measures such as mortality to report the clinical effectiveness of a treatment or intervention. Improvement science uses a variety of measures such as engagement, process and outcome measures.

An engagement measure would be used to assess how well an individual or hospital is interacting with a project and may include measures such as patient recruitment or data completeness. Process measures show whether steps proven to benefit patients are followed correctly [25]. They measure whether an action was completed—such carrying out a preoperative risk assessment or enrolment of a patient to an enhanced recovery pathway.

Measuring processes provide a clearer understanding of what we do. Understanding them can provide additional insight into variation in outcomes measures. By using process measures, the development of targeted interventions to improve them can be more straightforward than using outcome measures alone.

Outcome measures take stock not of the processes, but of the actual results of care. They are generally the most relevant measures for patients and the measures that providers most want to change [25]. Meaningful comparisons of outcomes within the health care system generally require risk adjustment—accounting for patient-associated factors before comparing outcomes across different patients, treatments, providers or populations [26]. The reasons for this are obvious, patients with a poorer health status are on average likely to have poorer outcomes. Risk adjustment aims to account for differences in intrinsic health risks that patient brings to their healthcare encounters. For comparing outcomes, it thus “levels the playing field,” ensuring that “apples are compared to apples, not oranges” [26].

The need for risk adjustment poses a potential problem for quality improvement initiatives. Risk adjustment requires a suitable model that has been calibrated and validated for the cohort it is being used for. Model development often requires a large cohort of patients that may not be available to single institution projects. For data to be used for improvement it must also be made available to local teams in a timely fashion. Waiting months or even years after collection for risk-adjusted outcomes to be available does not support improvement.

There is a range of data visualisation options available within improvement science. Some of these aim to allow continuous displays of risk-adjusted outcomes in a timely fashion, such as the visual life-adjusted display, which is discussed more later.

### Data Feedback

There is compelling evidence that clinicians in other healthcare fields can improve clinical outcomes after receiving feedback on their performance. The need for individual monitoring and reflection has increased with the introduction of revalidation in the UK. A 2012 Cochrane review reported that ‘feedback may be more effective when baseline performance is low, the source is a supervisor or colleague, it is provided more than once, it is delivered in both verbal and written formats, and when it includes both explicit targets and an action plan. In addition, the effect size varied based on the clinical behaviour targeted by the intervention’ [27].

However, anaesthetists often feel that there is a lack of credible performance/quality indicators available to them. A recent qualitative study of anaesthetists’ perceptions of individualised feedback of recovery data reported that they preferred: indicators which reflect areas of care that they felt they could control; graphical data presentation; longitudinal and comparative data reporting and personalised reporting [28•].

The multidisciplinary teamwork nature of anaesthesia suggests that system level data is needed to be able to enact organisational changes.

### Data Visualisation Techniques

To support quality improvement, time-series data is generally used. This allows the visualisation of change over time, to assess if processes and outcomes are improving following intervention.

The simplest example is a line graph, with time on the *x*-axis and the process or outcome of interest on the *y*-axis. To support monitoring and alert users to variation in results over time, there has been a variety of additional data displays developed. These include the run chart (which has the addition of a median line) and statistical process control chart (which has the addition of a mean line and control limits). It is beyond the scope of this article to discuss these in detail, but articles by McQuillan et al. [29••] and Benneyan et al. [30•] serve as an excellent introduction to their application and interpretation.

Displays have also been developed to allow the continuous visualisation of risk-adjusted outcomes. One example of this is the visual life-adjusted display (VLAD). The VLAD chart shows expected vs. observed outcomes and has been used in a variety of settings including paediatric and adult cardiac surgery, intensive care and trauma [31–35].

**Fig. 1:**
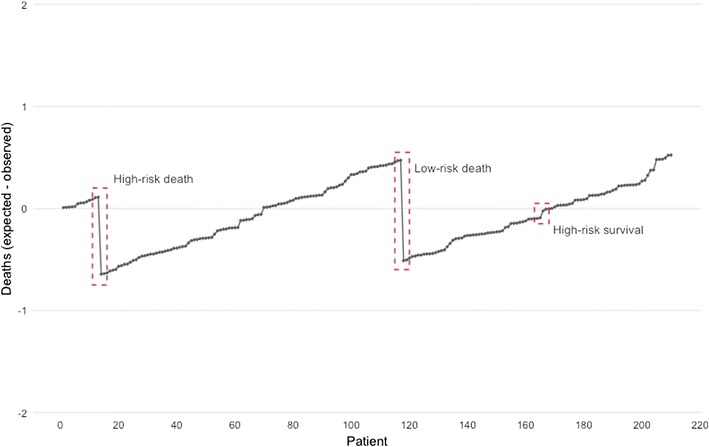
An example of a VLAD chart showing observed vs. expected deaths incorporating risk-adjustment

Figure 1 shows an example of a standard VLAD chart of the difference between the expected number of deaths over a series of 210 patients, calculated by summing the estimated risk for each episode, and the observed number of deaths. The trace rises with each survival (more so for high-risk episodes) and falls with each death (more so for low-risk episodes). In the example chart, the ‘high-risk death’ had a predicted mortality risk of 25%, compared to a predicted mortality risk of 1.6% for the ‘low-risk death’. The negative deflection is greater for a low-risk death compared to that of a high-risk. Over time, if observed outcomes matched expected outcomes, the VLAD chart will be hover around the zero point on the *y*-axis.

There are also other forms of display proposed that incorporate risk adjustment such as the risk-adjusted cumulative sum, risk-adjusted sequential probability ratio and risk-adjusted exponentially weighted moving average charts [36].

### Data Usage

Obtaining reliable measurements of a system is a necessary but not sufficient step in making improvement. Indeed, no association was found between participation in NSQIP and improved clinical outcomes in large clinical cohort of patients undergoing elective general/vascular surgery in the USA [21•]. This surprising finding may be explained by recent UK studies which have reported how little National Clinical Audit data gets used for practical improvement [37•]. A large multimethod study of the NHS found that ‘organisations were putting considerable time, effort and resources into data collection..[but] the degree to which this translated into actionable knowledge, and then into effective organisational responses, differed markedly between organisations’ [38]. An evidence scan by the Health Foundation reported that insufficient use of data was a barrier to implementation at every stage of the design, delivery and dissemination of improvement strategies in the NHS [39]. This failure to use data effectively represents a waste of both resources and opportunities.

The reasons why data may not be used to its full potential may lie with the data collection and feedback systems, the individuals receiving the data or the context in which the healthcare system is situated.

Making data ‘usable’ for improvement not only relies upon the criteria for effective feedback as described above, but also requires understanding the needs of the data’s users. Two often conflated but potentially conflicting needs are those of quality assurance (QA) and quality improvement (QI). Typically, QA identifies compliance with an agreed standard, whereas QI reflects iterative positive change towards best-practice [40]. In the UK, the Healthcare Quality Improvement Partnership (HQIP), the commissioner of National Clinical Audits, is currently collaborating with the Care Quality Commission (CQC), the healthcare regulator, to optimise their data for both QA and QI. This will likely culminate in a National Clinical Audit dashboard, which aims to reduce the data collection burden; co-localise audit results in the same place to enable hospitals to take a cross-cutting view of quality; increased dynamism and interactivity [41].

The choice of quality indicator clearly matters. Clinicians are naturally driven to improve patients’ outcomes, but process measures may provide more scope for improvement in the short term. The challenge of using reliable patient-reported outcome measures is well documented; a recent review concluded that they were best sued as ‘tin-openers’ (highlighting problems or areas of success) rather than ‘dials’ (quantifying change) [42].

Potential solutions are emerging to support data usage and include efficient trial designs such as that demonstrated by the EPOCH trial [43•]. This study aims to support clinicians in 90 UK hospitals use QI methods and NELA data to help implement a care pathway to nationally agreed standards, and is being evaluated by stepped wedge cluster randomised trial.

Finally, unintended consequences of reporting quality data should be anticipated as well as expected benefits. Individual level outcome data of surgeons in the UK has been met with some resistance, with concerns ranging from doubt over risk-adjustment processes to potential risks of gaming [44, 45]. A hospital ‘Report Card’ initiative for cardiac surgery in New York and Pennsylvania in the early 1990s resulted in surgeons selecting to operate on healthier patients compared to neighbouring states without report cards [46]. HQIP notes that the potential risks of their proposed new dashboard include over-simplification, duplication and gaming.

## Conclusions

Improvement Science is a developing field primarily concerned with maximising and sharing learning from QI projects. It aims to use stringent scientific method to support and analyse quality improvement initiatives. Large-scale perioperative data measurement and feedback systems are being introduced in both the USA and UK and represent opportunities for anaesthetists to work with multidisciplinary colleagues to help improve services and outcomes for surgical patients. Experience tells us that making the most of these opportunities is difficult; evaluating which approaches work, and in which contexts, seems an ever more urgent priority for healthcare commissioners, providers and patients.
